# Thread-Traction with a Sheath of Polypectomy Snare Facilitates Endoscopic Submucosal Dissection of Early Gastric Cancers

**DOI:** 10.1155/2016/9415497

**Published:** 2015-12-30

**Authors:** Hisatsugu Noda, Naotaka Ogasawara, Akira Koshino, Shouko Fukuta, Takuroh Nagoya, Hironori Hoshino, Kazuhiro Nagao, Tomoya Sugiyama, Yoshihiro Kondo, Yoshitsugi Ito, Shinya Izawa, Masahide Ebi, Yasushi Funaki, Makoto Sasaki, Kunio Kasugai

**Affiliations:** Department of Gastroenterology, Aichi Medical University School of Medicine, 1-1 Yazakokarimata, Nagakute, Aichi 480-1195, Japan

## Abstract

Although the thread-traction (TT) method has been found useful during endoscopic submucosal dissection (ESD) for early gastric cancers, the movement of the thread interferes with the movement of the endoscope, and the lesion can only be pulled to the mouth side. We have developed the novel TT method using a sheath of polypectomy snare (TTSPS). The TTSPS method enables free and independent movement of the thread and the endoscope and allows pulling the lesion towards the anal as well as oral side. The median dissection times, numbers of instances of arterial bleeding, and numbers of local injections into the submucosal layer were significantly lower for ESD with TTSPS than for conventional ESD. Countertraction ESD using the TTSPS method is straightforward, safe, easy, noninvasive, and cost effective, and it uses instruments readily available in most hospitals to enhance visualization of cutting lines. Therefore, the TTSPS method can be universally applied in conventional ESD.

## 1. Introduction

Endoscopic submucosal dissection (ESD) has replaced conventional endoscopic mucosal resection (EMR) [1, 2] as a standard therapy for early gastric neoplasms in Japan [3]. En bloc lesion resection can be achieved with ESD by using various types of knives and a number of modifications [3–8]. However, ESD is associated with several adverse events, such as bleeding and perforation, and therefore it requires more skill than EMR [9, 10]. During ESD, the mobility of the lesion increases as submucosal dissection proceeds, and attaining effective countertraction becomes difficult. To overcome this problem, various traction methods, such as the use of magnetic anchors [11], sinker-assisted ESD [12], the use of external grasping forceps [13], sheath-assisted countertraction [14, 15], and the pulley method [16], have been developed. The recently introduced thread-traction (TT) method [17] has been reported to be technically easier than other traction methods. The TT method ensures effective countertraction while maintaining the view of the dissected submucosal field. Koike et al. [17] reported that this allows efficient dissection, which reduces procedure time. However, during ESD with TT, the movement of the thread physically interferes with the movement of the endoscope, making it difficult to achieve the desired traction. Moreover, in the TT method, the lesion can be pulled only towards the mouth side but not towards the anal side.

To eliminate these shortcomings, we have developed a modification of the TT method, termed TT with a sheath of polypectomy snare (TTSPS), which enables performing the procedure without disturbing the movement of the thread by the endoscope and allows pulling the lesion towards either oral or anal side regardless of the lesion location. This study aimed to compare ESD with TTSPS and conventional ESD that did not employ the new traction system in terms of tumor size, en bloc resection rate, duration of the ESD procedure depending on tumor size and location, and complication rates.

## 2. Methods

### 2.1. Patients

Thirty-four consecutive patients with well- or moderately differentiated adenocarcinoma and adenoma, confirmed by histological evaluation of biopsy specimens obtained using forceps between May 2013 and December 2013, underwent conventional ESD that did not employ the new traction system. Fifty-four consecutive patients with well- or moderately differentiated adenocarcinoma and adenoma, confirmed by histological evaluation of biopsy specimens between January 2014 and March 2015, underwent ESD with TTSPS. This study was approved by the Ethics Committee of Aichi Medical University School of Medicine, and written informed consent was obtained from all patients to all study procedures. ESD was indicated for the following types of suspected lymph node-negative early gastric cancer: intramucosal differentiated-type adenocarcinoma without ulcers regardless of size, intramucosal differentiated-type adenocarcinoma with ulcers if ≤3 cm in size, or intramucosal undifferentiated-type adenocarcinoma without ulcers if ≤2 cm in size. Histopathological diagnoses were based on the Japanese Classification of Gastric Carcinoma. Clinical results in the two groups were compared retrospectively.

### 2.2. ESD Procedure

All patients were sedated with intravenous midazolam (Astellas Pharma Inc., Tokyo, Japan) (0.1 mg/kg) and pentazocine (Daiichi Sankyo Co., Tokyo, Japan) (15 mg), and sedation was maintained with intermittent injections of midazolam (10–20 mg) during ESD. We performed ESD using a single-channel (GIF-Q260J; Olympus, Tokyo, Japan) or double-channel (GIF-2TQ260M; Olympus) video endoscope. A short, disposable, transparent attachment (D-201-10704; Olympus) was attached to the endoscopic tip to improve the visualization of the lesion where necessary. A flexible overtube (Top Co., Ltd., Tokyo, Japan) enabled repeated insertion and retrieval of the endoscope. After indigo carmine dye (Daiichi Sankyo Co., Tokyo, Japan) staining, marker dots were placed about 5 mm outside the lesion margin using a flush knife (DK2618JB; Fujifilm, Tokyo, Japan) operating in the forced coagulation mode at the power of 30 W delivered by an electrosurgical unit (VIO300D; ERBE, Tübingen, Germany). Epinephrine (Daiichi Sankyo Co., Tokyo, Japan) (0.025%) and indigo carmine dye (0.005%) diluted in hyaluronic acid (Johnson and Johnson Co., Tokyo, Japan) were injected around the lesion to lift the submucosa, and one or two small holes were made for knife insertion. The ESD procedure was performed with an IT knife, flush knife, SB knife, or Clutch Cutter as determined by the endoscopist. On the day of ESD, intravenous omeprazole (20 mg/day), sodium alginate (90 mL/day), and aluminum hydroxide (30 mL/day) were started. After completion of ESD, a nasogastric tube (Salem Sump tube; Covidien, Dublin, Ireland) was inserted in all patients to promptly detect instances of bleeding of iatrogenic ulcers.

### 2.3. ESD with Thread-Traction with a Sheath of Polypectomy Snare (ESD with TTSPS)

**Figure 1:**
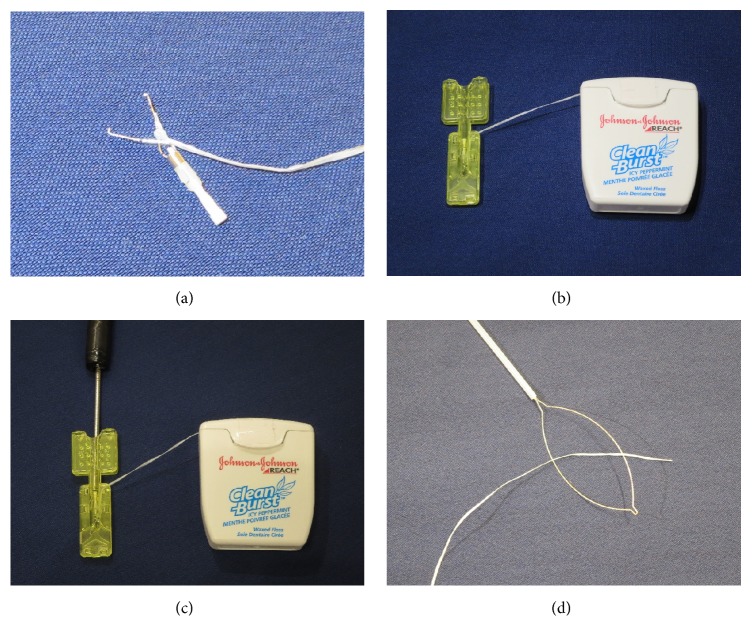
Preparation for thread-traction using a sheath of polypectomy snare (part 1). One end of a thread is tied to the claw of a clip (a), and the clip with the thread is reinstalled into the clip case (b). After the rotatable clip-fixing device is inserted into the channel of the endoscope, the clip with the thread is attached to a rotatable clip-fixing device (c). The other end of the thread is passed through the loop of a polypectomy snare (d).

**Figure 2:**
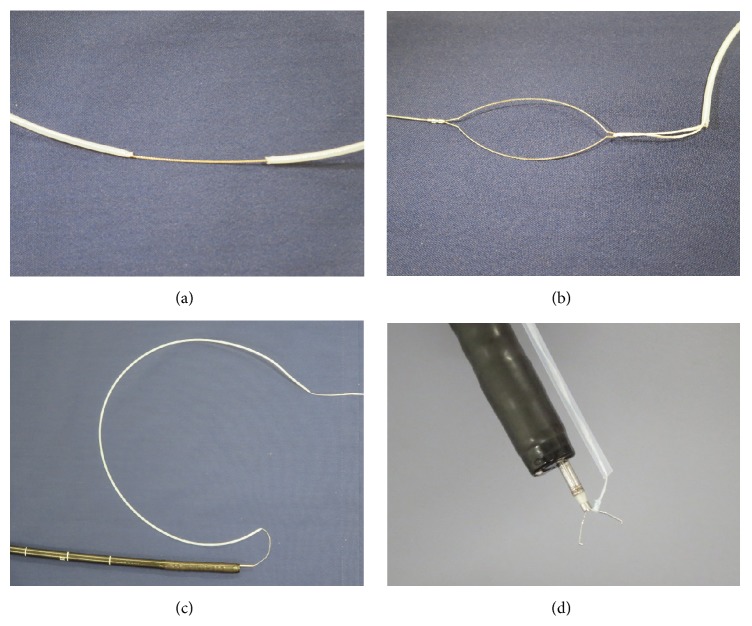
Preparation for thread-traction using a sheath of polypectomy snare (part 2). The sheath of the snare is cut at about 60 cm with scissors (a). The polypectomy snare holding the thread is gradually retrieved, and the thread completely passes through the sheath of the snare (b and c). The sheath of the snare is positioned close to the clip (c). The endoscope and the sheath containing the thread are simultaneously inserted into the stomach (d).

**Figure 3:**
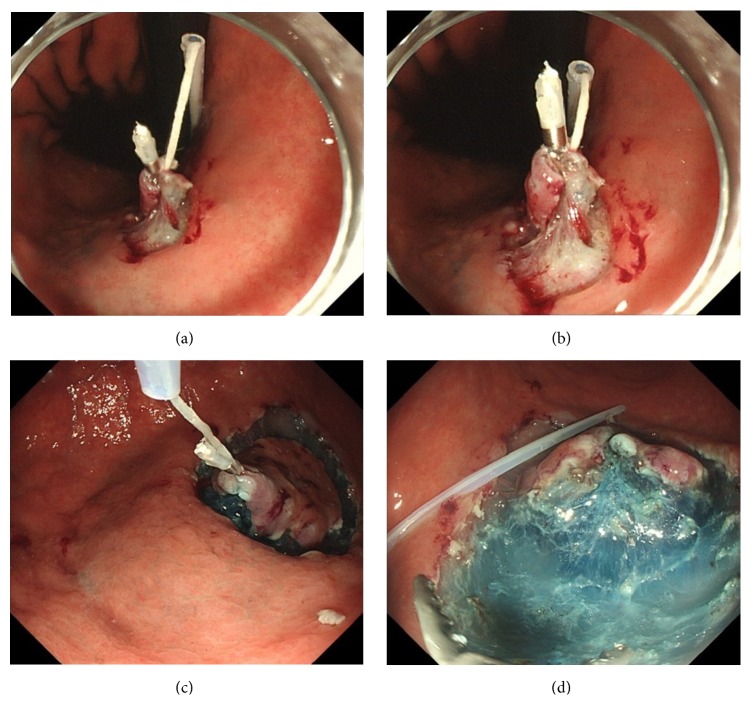
After circumferential mucosal incision, the clip with the thread covered by the sheath is attached to the edge of the lesion, including both the mucosal and submucosal layers (a). The thread-traction methods using a sheath of polypectomy snare (TTSPS) allow independent movement of the thread and the endoscope, and traction force can be easily controlled without interfering with the movement of the endoscope (a and b). The lesion can be pulled not only towards the oral side (c) but also towards the anal side by positioning the sheath over the anal side of the lesion (d). In the TTSPS method, countertraction can be easily applied to the anal side of the tumor, and the submucosal layer of the tumor is clearly visible when the tumor is located in the antrum (c and d).

A clip with thread was composed of a rotatable clip-fixing device (Olympus Medical Systems, Co.), a short clip (HX610-090S; Olympus Medical Systems, Co.), and a thread (Unflavored Waxed Floss, Johnson and Johnson Co., Tokyo, Japan) of about 1 m in length. One end of the thread was tied to the claw of the clip (Figure 1(a)), and the clip with the thread was reinstalled into the clip case (Figure 1(b)). After the rotatable clip-fixing device is inserted into the channel of the endoscope, the clip with the thread is attached to a rotatable clip-fixing device (Figure 1(c)). The other end of the thread was passed through the loop of a polypectomy snare (CAPTIVATOR 27 mm, Boston Scientific, USA) (Figure 1(d)), and the sheath of the snare was cut at about 60 cm with scissors (Figure 2(a)). The polypectomy snare holding the thread was then completely pulled through the sheath (Figure 2(b)). Thereby, the edge of the sheath approached the rotatable clip-fixing device inserted into the endoscope and the thread completely passed through the sheath (Figure 2(c)). Finally, the endoscope and the sheath containing the thread were inserted into the stomach as a single unit (Figure 2(d)). After performing a circumferential mucosal incision, the clip with the thread covered by the sheath was attached to the edge of the lesion, including both mucosal and submucosal layers (Figure 3(a)). As a result, the thread could be moved without interfering with the movement of the endoscope, and the traction force could be easily controlled (Figures 3(a) and 3(b)). Because the entire length of the thread attached to the clip was covered by the sheath, which enabled free movement of the thread, the thread could be moved independently during the ESD procedure. Moreover, this setup allowed pulling the thread both towards the oral (Figure 3(c)) and anal side by repositioning the sheath accordingly (Figure 3(d)). The sheath covering the thread could also be moved back and forth independently, providing the means of easily controlling the force of traction to visualize the submucosal layer cutting line and of applying appropriate amounts of tension to the submucosa regardless of the lesion location. Thus, the TTSPS method allowed applying countertraction toward the anal side of the tumor, and the submucosal layer of the tumor was easy to visualize when the tumor was located in the antrum (Figures 3(c) and 3(d)). In addition, this also extended the range of movement of knife devices, which could be manipulated more independently during the ESD procedure.

### 2.4. Comparison between Conventional ESD and ESD Using the TTSPS Method

Dissection time, number of instances of arterial bleeding, and number of local injections into the submucosal layer of the tumor were compared between the conventional ESD group (*n* = 34) and the group with countertraction ESD using the TTSPS method (*n* = 54) for early gastric neoplasms. Dissection time was defined as the time interval between completion of the circumferential mucosal incision and completion of tumor dissection. For ESD with TTSPS, dissection time included the time required for grasping the specimen with the clip with attached thread. Local injections were performed to dissect the submucosa safely under good endoscopic view. The number of local injections and number of occurrences of arterial bleeding were counted in the period between completion of the circumferential mucosal incision and completion of tumor dissection. Complications such as perforation during or after ESD and post-ESD bleeding were also evaluated in the two groups.

### 2.5. Statistical Analysis

Data were analyzed using the Fisher exact test, the chi-square (*χ* ^2^) test, or the Mann-Whitney *U* test for differences between the groups. A *p* value < 0.05 was considered to indicate statistical significance.

## 3. Results

There were no significant differences in the patients' characteristics between the two groups (Table 1). Furthermore, the groups did not differ with respect to the location and differentiation of tumors, their size, and en bloc resection rates (Table 2). The median dissection time differed significantly for all lesions between conventional ESD (90 min, range: 30–320 min) and ESD with TTSPS (60 min, range: 15–160 min) (*p* = 0.015) (Table 3). The median number of occurrences of arterial bleeding was significantly lower for ESD with TTSPS (2, range: 0–7) than for conventional ESD (3, range: 0–25) (*p* = 0.015) (Table 3). The median number of local injections significantly differed between the two groups (conventional ESD: 10, range: 3–51; ESD with TTSPS: 8, range: 1–27; and *p* = 0.04) (Table 3). In the conventional ESD group, perforation occurred in 2 patients, and 3 patients underwent endoscopic treatment for delayed bleeding after the ESD procedure (Table 4). In the ESD with TTSPS group, perforation occurred in only 1 patient, and another patient required endoscopic treatment because of delayed bleeding (Table 4). There were no significant differences in the rates of complications between conventional ESD and ESD with TTSPS (Table 4). All the patients, except for 3 patients with perforation, were discharged within 8 days.

## 4. Discussion

Gastric neoplastic lesions, including early gastric cancers, are currently resected using ESD [3, 9, 18]. However, ESD is associated with an increased incidence of complications, such as bleeding and perforation [18], and the procedure is technically more difficult than conventional EMR [9, 10]. Thus, delayed bleeding after ESD occurs in about 6% of patients, whereas perforation, which is the most critical complication, occurs in about 4% of patients during the ESD procedure [3]. Complications of ESD frequently arise because the dissected submucosa cannot be stabilized or visualized. These difficulties lead to inaccurate identification of the cutting line and inadvertent cutting of submucosal vessels, which causes bleeding, as well as to underestimation of the submucosal layer depth, which leads to perforation [12]. Stabilizing and visualizing the submucosa by applying appropriate tension can reduce the incidence of complications. Therefore, noninvasive tools and methods that facilitate direct visualization of the submucosal layer are required to reduce complications during and after ESD.

Various traction devices and techniques have been designed to alleviate the above-mentioned shortcomings of ESD and reduce the procedure time, including percutaneous traction-assisted EMR (PTA-EMR) [19], magnetic anchors [11], sinker-assisted ESD [12], R-scope (an endoscope with 2 instrument channels produced by Olympus) [20], a double-endoscope intraluminal procedure that requires participation of two endoscopists [21, 22], the use of external grasping forceps [13], sheath-assisted countertraction [14, 15], and the pulley method [16]. Overall, these tools have been proven useful in facilitating ESD. However, PTA-EMR requires a laparoscopic port with a trocar, and the magnetic anchor system requires a large, expensive control device that is not yet available for clinical use. In sinker-assisted ESD, the traction direction is controlled by changing the position of the patient, and, although this technique was found to be effective for colorectal neoplasms, its application to gastric neoplasms is limited by the necessity to keep the patient in the left decubitus position during the procedure. R-scopes have two instrument channels, one of which is used to move a forceps vertically to grasp the lesion while the other is utilized to move a cutting knife horizontally to perform dissection. However, the range of movement of the knife may be insufficient for successful resection of the submucosal layer because the lesion should be tightly held by the forceps. The sheath-assisted countertraction method requires a specialized double-channel video endoscope, which is not readily available in many institutions. Finally, in the pulley method, the thread may interfere with the endoscope.

The TT method, with its simple procedure and no special requirements to the endoscopy equipment, has proven to be very useful. However, in this approach, the movement of the thread can interfere with the movement of the endoscope because the thread and the body of the endoscope rub against each other, resulting in inability to achieve appropriate traction. Another constraint is the fact that the lesion can only be pulled to the mouth side but not to the anal side. Therefore, it is difficult to control the force and direction of traction precisely within this approach, and, as a result, its applicability can be limited by the tumor location in some cases. In contrast, the new TTSPS method eliminates the interference between the thread and the endoscope, and the traction force can be easily controlled without affecting the movement of the endoscope regardless of the lesion location. This independence in movement of the thread attached to the clip is achieved by using a sheath that completely covers the thread. Furthermore, the lesion can be pulled not only towards the oral side but also towards the anal side by positioning the sheath over the anal side of the lesion. The sheath can also move independently, which allows controlling the traction force by moving the sheath back and forth regardless of lesion location. The practical implementation of this approach allowed free and independent movement of both the thread to lift the submucosal layer and the endoscope and dissecting devices to perform submucosal dissection.

Our study showed that the TTSPS approach significantly reduced the procedure time, the number of instances of arterial bleeding, and the number of local injections compared with conventional ESD. Although the number of complications also tended to be lower for ESD with TTSPS, the differences did not reach the level of statistical significance. These results suggest that ESD using TTSPS is safer and less invasive than conventional ESD and that the new TTSPS method should be universally applicable to standard ESD. Moreover, we found that ESD with countertraction provided by the TTSPS approach is technically simpler and therefore less time consuming regardless of lesion location. ESD with TTSPS facilitated identification and direct visualization of gastric lesions, all of which were resected en bloc.

After testing a number of sheaths, we have found that the sheath of a polypectomy snare (Captivator 27 mm, Boston Scientific, USA) produced the best results in terms of appropriate traction because of its proper elasticity and flexibility, and passing the thread through the polypectomy snare sheath was easy. Furthermore, this method requires neither special instruments nor unusual devices because virtually all institutions performing gastrointestinal endoscopy already have polypectomy snares or can easily obtain them.

In addition to facilitating direct visualization of the submucosal layer cutting line and allowing applying appropriate amounts of tension to the submucosa, the TTSPS method simplifies achieving hemostasis and manipulating blood vessels, which helps to avoid serious complications. Furthermore, TTSPS is cost-effective and practical, and it can be immediately introduced into clinical practice. The tools designed for various types of ESD discussed above are relatively difficult to use as considerable training and experience are required before they can be effectively applied. In contrast, ESD using TTSPS is straightforward and technically conventional, and therefore it does not require specific practice and training.

In conclusion, we have developed a noninvasive, simple, safe, and inexpensive method that enables direct visualization of the cutting line and thus facilitates ESD of selected neoplastic lesions in the stomach. Countertraction ESD using TTSPS required no specialized equipment and was technically simpler and therefore less time consuming regardless of lesion location. Importantly, endoscopists of any skill level can safely, efficiently, and completely remove early gastric cancers using ESD with internal traction provided by the TTSPS approach. This makes the TTSPS method universally applicable in conventional ESD.

## Figures and Tables

**Table 1:** Patients' characteristics.

	Conventional ESD	ESD with TTSPS	*p *value
Sex (male : female)	26 : 8	48 : 6	NS

Median age (yrs) (range)	75 (34–91)	73 (46–86)	NS

Hypertension (present : absent)	22 : 12	32 : 22	NS

Diabetes mellitus (present : absent)	6 : 28	10 : 44	NS

Liver disease (present : absent)	4 : 30	0 : 54	NS

Hemodialysis (present : absent)	0 : 34	0 : 54	NS

Other comorbidities (present : absent)	4 : 30	0 : 54	NS

Rate of usage of anticoagulant and/or antiplatelet drugs (%)	11.8 (4/34)	22.2 (12/54)	NS

ESD: endoscopic submucosal dissection; NS: not significant; and TTSPS: thread-traction method using a sheath of polypectomy snare.

**Table 2:** Characteristics of the tumors.

	Conventional ESD	ESD with TTSPS	*p *value
Number of lesions	34	54	

Location (U : M : L)	4 : 16 : 14	6 : 26 : 22	NS

Histological type	Adenoma: 10 Diff.: 22 Undiff.: 2	Adenoma: 12 Diff.: 36 Undiff.: 6	NS

Depth (mucosal : submucosal)	26 : 8	36 : 18	NS

Macroscopic type (depressed : nondepressed)	14 : 20	26 : 28	NS

Mean resected size (mm) (range)	30 (14–60)	34 (16–55)	NS

En bloc resection rate (%)	97.1 (33/34)	100 (54/54)	NS

ESD: endoscopic submucosal dissection; NS: not significant; TTSPS: thread-traction method using a sheath of polypectomy snare; U: fundus; M: corpus; L: antrum and pylorus; diff.: differentiated adenocarcinoma; and undiff.: undifferentiated adenocarcinoma.

**Table 3:** Comparisons between conventional ESD and ESD with TTSPS.

	Conventional ESD	ESD with TTSPS	*p *value
Median dissection time (min) (range)	90 (30–320)	60 (15–160)	0.015
Median number of incidences of arterial bleeding (range)	3 (0–25)	2 (0–7)	0.015
Median number of local injections (range)	10 (3–51)	8 (1–27)	0.04

ESD: endoscopic submucosal dissection; NS: not significant; and TTSPS: thread-traction method using a sheath of polypectomy snare.

**Table 4:** Comparisons of complications between conventional ESD and ESD with TTSPS.

	Conventional ESD	ESD with TTSPS	*p *value
Post-ESD bleeding (%) (present : absent)	3 : 31 (8.8%)	1 : 53 (1.9%)	NS
Perforation (%) (present : absent)	2 : 32 (5.9%)	1 : 53 (1.9%)	NS

ESD: endoscopic submucosal dissection; NS: not significant; and TTSPS: thread-traction method using a sheath of polypectomy snare.

## References

[1] Soetikno R. M., Gotoda T., Nakanishi Y., Soehendra N. (2003). Endoscopic mucosal resection. *Gastrointestinal Endoscopy*.

[2] Tanabe S., Koizumi W., Mitomi H. (2002). Clinical outcome of endoscopic aspiration mucosectomy for early stage gastric cancer. *Gastrointestinal Endoscopy*.

[3] Gotoda T. (2007). Endoscopic resection of early gastric cancer. *Gastric Cancer*.

[4] Hirasaki S., Tanimizu M., Moriwaki T. (2004). Efficacy of clinical pathway for the management of mucosal gastric carcinoma treated with endoscopic submucosal dissection using an insulated-tip diathermic knife. *Internal Medicine*.

[5] Kodashima S., Fujishiro M., Yahagi N., Kakushima N., Omata M. (2006). Endoscopic submucosal dissection using flexknife. *Journal of Clinical Gastroenterology*.

[6] Oka S., Tanaka S., Takata S., Kanao H., Chayama K. (2012). Usefulness and safety of SB knife Jr in endoscopic submucosal dissection for colorectal tumors. *Digestive Endoscopy*.

[7] Toyonaga T., Man-I M., Fujita T. (2010). The performance of a novel ball-tipped Flush knife for endoscopic submucosal dissection: a case-control study. *Alimentary Pharmacology and Therapeutics*.

[8] Ono H., Hasuike N., Inui T. (2008). Usefulness of a novel electrosurgical knife, the insulation-tipped diathermic knife-2, for endoscopic submucosal dissection of early gastric cancer. *Gastric Cancer*.

[9] Onozato Y., Ishihara H., Iizuka H. (2006). Endoscopic submucosal dissection for early gastric cancers and large flat adenomas. *Endoscopy*.

[10] Oda I., Gotoda T., Hamanaka H. (2005). Endoscopic submucosal dissection for early gastric cancer: technical feasibility, operation time and complications from a large consecutive series. *Digestive Endoscopy*.

[11] Kobayashi T., Gotohda T., Tamakawa K., Ueda H., Kakizoe T. (2004). Magnetic anchor for more effective endoscopic mucosal resection. *Japanese Journal of Clinical Oncology*.

[12] Saito Y., Emura F., Matsuda T. (2005). A new sinker-assisted endoscopic submucosal dissection for colorectal cancer. *Gastrointestinal Endoscopy*.

[13] Imaeda H., Iwao Y., Ogata H. (2006). A new technique for endoscopic submucosal dissection for early gastric cancer using an external grasping forceps. *Endoscopy*.

[14] Hijikata Y., Ogasawara N., Sasaki M. (2010). Endoscopic submucosal dissection with sheath-assisted counter traction for early gastric cancers. *Digestive Endoscopy*.

[15] Hijikata Y., Ogasawara N., Sasaki M. (2012). Endoscopic submucosal dissection with sheath-assisted counter traction using a novel sheath for early gastric cancers. *Hepato-Gastroenterology*.

[16] Li C.-H., Chen P.-J., Chu H.-C. (2011). Endoscopic submucosal dissection with the pulley method for early-stage gastric cancer (with video). *Gastrointestinal Endoscopy*.

[17] Koike Y., Hirasawa D., Fujita N. (2015). Usefulness of the thread-traction method in esophageal endoscopic submucosal dissection: randomized controlled trial. *Digestive Endoscopy*.

[18] Toyokawa T., Inaba T., Omote S. (2012). Risk factors for perforation and delayed bleeding associated with endoscopic submucosal dissection for early gastric neoplasms: analysis of 1123 lesions. *Journal of Gastroenterology and Hepatology*.

[19] Kondo H., Gotoda T., Ono H. (2004). Percutaneous traction-assisted EMR by using an insulation-tipped electrosurgical knife for early stage gastric cancer. *Gastrointestinal Endoscopy*.

[20] Yonezawa J., Kaise M., Sumiyama K., Goda K., Arakawa H., Tajiri H. (2006). A novel double-channel therapeutic endoscope (‘R-scope’) facilitates endoscopic submucosal dissection of superficial gastric neoplasms. *Endoscopy*.

[21] Kuwano H., Mochiki E., Asao T., Kato H., Shimura T., Tsutsumi S. (2004). Double endoscopic intraluminal operation for upper digestive tract diseases: proposal of a novel procedure. *Annals of Surgery*.

[22] Uraoka T., Kato J., Ishikawa S. (2007). Thin endoscope-assisted endoscopic submucosal dissection for large colorectal tumors (with videos). *Gastrointestinal Endoscopy*.

